# The diagnostic value of D-dimer with simplified Geneva score (SGS) pre-test in the diagnosis of pulmonary embolism (PE)

**DOI:** 10.1186/s13019-020-01222-y

**Published:** 2020-07-20

**Authors:** Zhihui Fu, Xibin Zhuang, Yueming He, Hong Huang, Weifeng Guo

**Affiliations:** Department of Respiratory, Quanzhou First Hospital Affiliated to Fujian Medical University, No. 248-252 Dongjie Street, Quanzhou, 362000 Fujian Province China

**Keywords:** D-dimer, Pulmonary embolism (PE), Simplified Geneva score (SGS), Cutoff point

## Abstract

**Background:**

Pulmonary embolism (PE) is the third most common cardiovascular syndrome with an average annual incidence rate of 77 per 100,000 population in the worldwide. The diagnose algorithms for suspected PE are generally include clinical scoring assessment and plasma D-dimer evaluation, patients with high risk of PE require computed tomographic pulmonary angiogram (CTPA) detection for confirmation.

**Methods:**

In this retrospective analysis, 1035 patients with suspected PE were recruited. All the patients were clinically received simplified Geneva score (SGS) pre-test, determination of plasma D-dimer level, and CTPA detection. All enrolled patients were grouped according to the CTPA results: PE patients and non-PE patients. Then, receiver operating characteristic (ROC) curve were constructed to determine the optimal D-dimer cutoff point value which is based on Yonden’s index (YI).

**Results:**

294 (28.4%) patients were confirmed with PE and 741(71.6%) individuals were regarded as non-PE cases by CTPA detection. Using the SGS pre-test, 829 (80.1%) patients were classified PE-unlikely (SGS ≤ 2) and 206 (19.9%) patients were PE-likely (SGS ≥ 3). Patients with D-dimer levels above 1.96 mg/L had a significant risk to suffer from PE (area under curve (AUC), 0.707; 95% CI, 0.678–0.735; *p* < 0.05). Meanwhile, in patients with SGS ≥ 3, the D-dimer cutoff point value moved to 2.2 mg/L (AUC, 0.644; 95% CI, 0.574–0.709; *p <* 0.05).

**Conclusion:**

D-dimer test in combination with SGS pre-test could improve the accuracy of PE diagnosis. Patients with D-dimer levels over 1.96 mg/L (4 times of the normal level) have a significant risk for PE. In patients with SGS ≥ 3, the D-dimer cutoff point concentration for PE risk moves to the levels of 2.2 mg/L.

## Background

Pulmonary embolism (PE) is a fatal condition which is induced by thromboembolus occluding the main pulmonary artery or its branches [1]. It is the third most common acute cardiovascular syndrome after myocardial infarction and stroke [2]. In American, it is estimated that venous thromboembolism, including PE and deep-vein thrombosis (DVT), may induce300 000 deaths every year using a modeling method [3]. Owing to using of more effective interventions and better adherence to guidelines, the mortality rate of acute PE has been reducing in many areas of the world [4–7]. However, it is also reported that there is an over-diagnosis in the diagnosis of PE in the current [8], this may in turn induce a fake decline of mortality rate to a certain extent. We consider that improving efficiency of PE detection methods and optimizing the detection process would be great benefit for the clinical outcomes of acute PE.

dimerA highly sensitive D-dimer test is available for venous thromboembolism diagnosis by exclusion without further testing [9]. Less sensitive, but more specific D-dimer detection exhibits advantages in identifying the patients with low thromboembolism risk [10]. Recent research has indicated that patients with the levels of D-dimer higher than 2.15 mg/L have a notable increased risk of PE [11]. It is hinted that the role of D-dimer on PE diagnosis test should be expanded, except for exclusion diagnosis of PE. According to the ESC guidelines, CTPA is the clinical “gold standard” for the diagnosis of PE [12].

The Geneva score is a clinical prediction rule to assess PE pre-test probability [12]. The original Geneva score was developed by Jacques Wicki et al. in 2001 [13]. To enhance clinical applicability and practicability, it has been simplified [14, 15]. Due to the limited sensitivity in the diagnosis of PE [16], the Geneva score is suggested to use in early assessment of PE clinical (pre-test) probability [12]. In present study, a retrospective analysis of 1035 cases of patients with suspected PE was carried out, and all the patients were performed with simplified Geneva score (SGS) assessment, D-dimer levels evaluation, and CTPA detection. We aimed to determine the D-dimer cutoff point for PE risk in patients with pre-test of SGS, and the patients with a D-dimer levels higher than that should be accepted further CTPA detection coercively.

## Materials and methods

### Patients

1035 patients with clinical suspected PE in the Quanzhou First Hospital Affiliated to Fujian Medical University from Jan, 2015 to Jun, 2019 were retrospectively analyzed. All the patients were accepted with SGS pre-test, D-dimer measurement, and CTPA. According to the results of CTPA, patients were divided into two groups: PE group and non-PE group. In addition, the following clinical information were collected from all the individuals: lung cancer, hypertension, diabetes, coronary disease, fibrinogen, morphological blood analysis, levels of C-reaction protein (CRP) and so on. Patients with missing or inconclusive statistical information were excluded. This study was approved by the Ethics Committee of Quanzhou First Hospital Affiliated to Fujian Medical University. Informed consent was obtained.

### Simplified Geneva score (SGS)

Simplified Geneva score (SGS) was calculated in accordance with the items in Table 1. In detail, the SGS assessment items included Age > 65, previous DVT or PE, surgery or fracture within one-month, active malignant condition, unilateral lower limb pain, hemoptysis etc. Most of the survey items just required a simple choice (yes/on) to make an easy and efficient assessment. Each item of the SGS had the same weight. An SGS ≤ 2 was presented as PE-unlikely, and an SGS ≥ 3 was regarded as PE-likely.

**Table 1:** Items for calculating Geneva score in original and simplified version are presented

Items	Original Version	Simplified Version
Age > 65	1	1
Previous DVT or PE	3	1
Surgery or fracture within one month	2	1
Active malignant condition	2	1
Unilateral lower limb pain	3	1
Hemoptysis	2	1
Heart rate		
75–94 b.p.m.	3	1
≥ 95 b.p.m.	5	2
Pain on deep palpation of lower limb and unilateral oedema	4	1
Clinical probability		
PE-unlikely	0–5	0–2
PE-likely	> 5	> 2

*DVT* deep vein thrombosis, *PE* pulmonary embolism, *b.p.m.* beat per minute

### Plasma D-dimer evaluation and CTPA

Plasma D-dimer levels were detected by immunoturbidimetry using ACL TOP 700 system (Beckman Coulter Inc., Fullerton, CA, USA) according to the manufacturer’s instructions. Concentration of D-dimer< 0.5 mg/L was regarded as normal level. In a routine process, patients with normal D-dimer level did not receive any further PE testing, while patients with positive D-dimer levels underwent CTPA detection. However, in present study, all enrolled patients were performed with CTPA detection (Light Speed VCT; GE Healthcare, USA) according to “pulmonary embolism” method. Patients with positive CTPA results were subjected to anticoagulant therapy. All PE-suspected patients were adjudicated by at least two experts until the final decision. All patients were followed up for 3 months.

### Statistical analysis

Data in present study were analyzed by using the MedCalc Statistical Software version 15.2.2 (MedCalc Software bvba, Ostend, Belgium). The purposed cutoff point of D-dimer for PE diagnosis was determined by receiver operating characteristic (ROC) curve. Youden’s index (YI = sensitivity + specificity – 1) of each point in ROC curve was evaluated to determine the cutoff point value with optimal sensitivity and specificity. Categorical variables were presented as number and percentage, and continuous variables were presented as mean ± SD (standard deviation). Comparisons between categorical variables were performed using Chi-square (χ^2^) test. Comparisons between continuous variables were performed using one-way analysis of variance (ANOVA) test. *P* < 0.05 was regarded as statistically significant difference.

## Results

### Analysis of general and clinical information for all patients

**Fig. 1:**
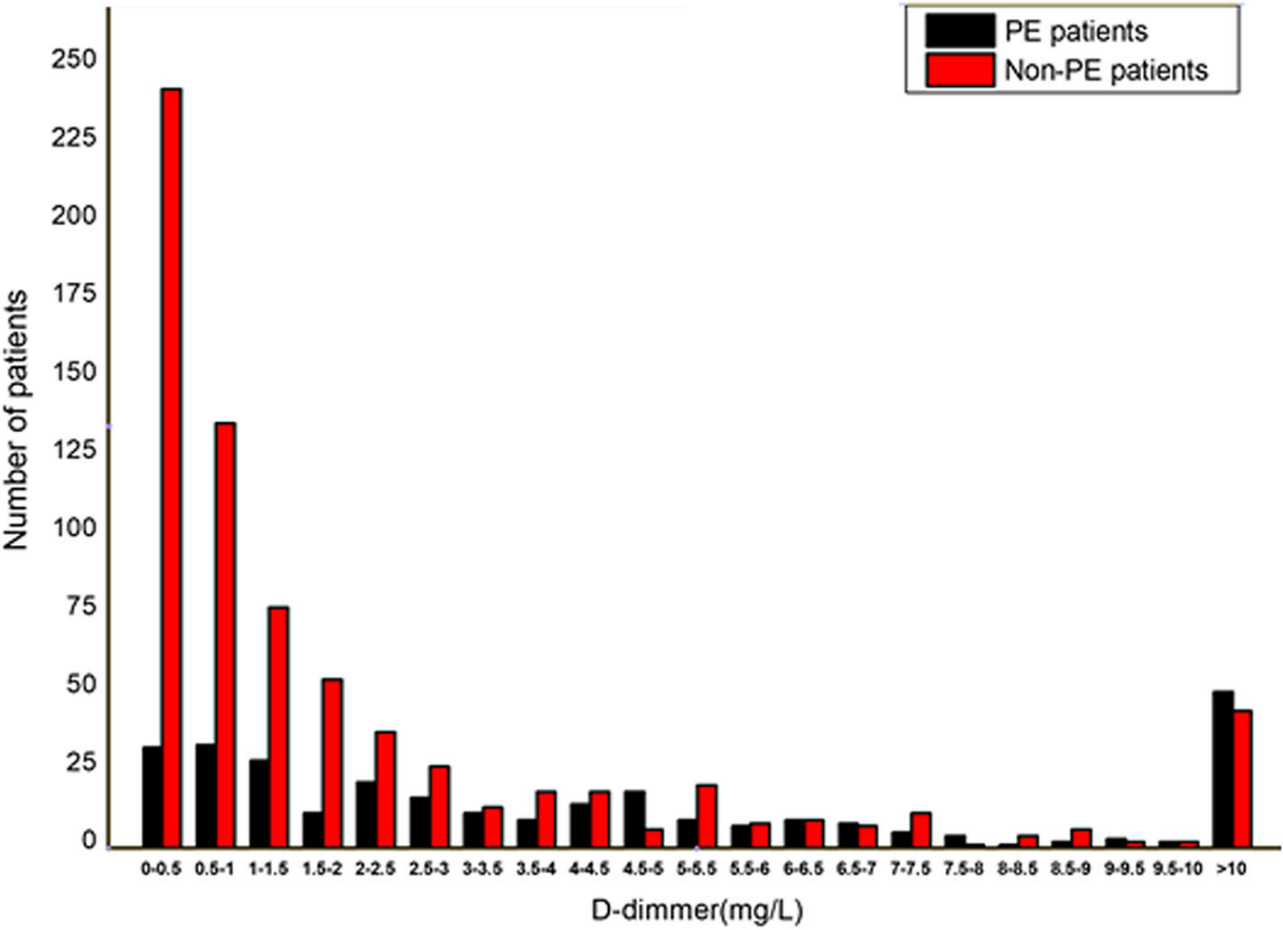
The frequency for PE and non-PE patients classified by D-dimer range. PE = pulmonary embolism

In this study, the enrolled patients consisted of 423(40.87%) females and 612(59.13%) males, the average age was 67.36 ± 9.79 years. Patients were divided into PE group (294 patients, 28.41%) and non-PE group (741 patients, 71.59%) according to the CTPA results. Among them, 68 patients (6.57%) were suffered from lung cancer of which pathological type mainly were adenocarcinoma and small cell cancer. It was found that patients with lung cancer were more likely to suffer from PE than those without lung cancer (*p* = 0.02). In addition, cough was the related factors for PE (*p =* 0.02), but other clinical symptoms including chest pain (*p* = 0.74), hemoptysis (*p* = 0.58), and dyspnea (*p* = 0.08) were not. The plasma D-dimer levels in PE patients (6.61 ± 11 mg/L) were significantly higher than those in non-PE patients (2.89 ± 5.99 mg/L, *p* < 0.001). The general and clinical information of all patients were presented in Table 2. The frequency of patients classified by D-dimer range was shown in Fig. 1.

**Table 2:** Clinical information of the PE and non-PE patients

Variates	Total	PE patients	Non-PE patients	*p*
	(*n =* 1035)	(*n* = 294)	(*n* = 741)	
Age (years)	67.36 ± 9.79	67.27 ± 9.47	67.4 ± 9.92	0.84
Gender				
Male n (%)	612 (59.13)	167 (56.8)	445 (60.05)	0.51
Female n (%)	423 (41.07)	127 (43.2)	296 (39.95)	
Lung cancer n (%)	68 (6.57)	28 (9.52)	40 (5.4)	0.02
Pulmonary infection n (%)	377 (36.43)	100 (34.01)	277 (37.38)	0.34
Hypertension n (%)	418 (40.39)	110 (37.41)	308 (41.57)	0.25
Diabetes n (%)	154 (14.88)	35 (11.9)	119 (16.06)	0.11
Coronary disease n (%)	71 (6.86)	14 (4.76)	57 (7.69)	0.12
Dyspnea n (%)	559 (54)	172 (58.5)	387 (52.23)	0.08
Chest pain n (%)	124 (11.98)	37 (12.59)	87 (11.74)	0.74
Cough n (%)	360 (34.78)	85 (28.91)	275 (37.11)	0.02
Hemopthysis n (%)	50 (4.83)	12 (4.08)	38 (5.13)	0.58
WBC	8.62 ± 6.71	9.00 ± 9.85	8.47 ± 4.95	0.26
HGB	125.22 ± 23.39	126.69 ± 21.67	124.63 ± 24.02	0.46
Neutr.	8.63 ± 6.71	9 ± 9.85	8.47 ± 4.95	0.26
PLT	232.66 ± 96.36	226.76 ± 89.76	234.99 ± 98.84	0.22
CRP	37.47 ± 56.05	38.07 ± 58.00	35.82 ± 50.33	0.61
FIB	3.49 ± 1.17	3.33 ± 1.06	3.56 ± 1.21	0.01
D-dimer (mg/L)	3.94 ± 7.92	6.61 ± 11	2.89 ± 5.99	< 0.001
SGS	1.72 ± 0.95	1.87 ± 0.96	1.66 ± 0.93	0.002

*PE* pulmonary embolism, *n* number, *WBC* white blood cell, *HGB* hemoglobin, *Neutr* neutrophil, *PLT* blood platelet, *CRP* C-reaction protein, *FIB* Fibrinogen, *SGS* Simplified Geneva score

### ROC curve to determine D-dimer proposed cutoff point value for PE diagnosis

**Fig. 2:**
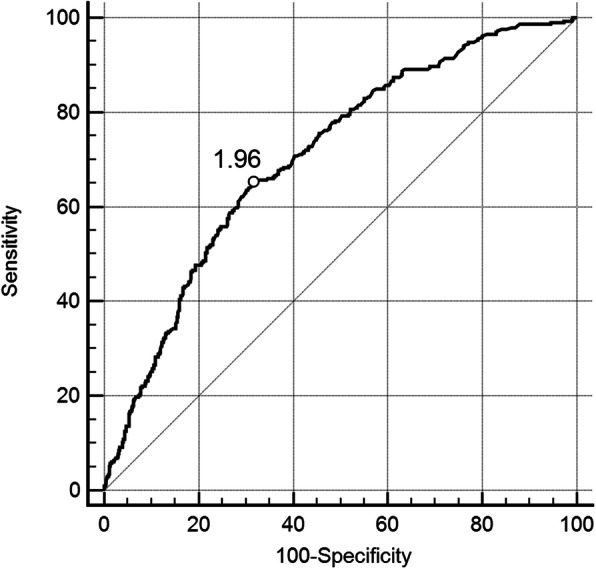
Receiver operating characteristic (ROC) curve for PE diagnosis by D-dimer evaluation. All patients were included (*n* = 1035). PE = pulmonary embolism

**Fig. 3:**
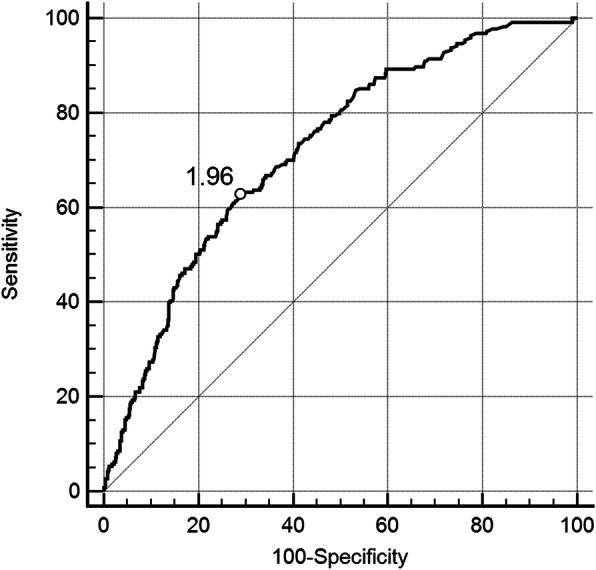
ROC curve for PE diagnosis by D-dimer evaluation. Patients with SGS ≤ 2 were included (*n* = 829). PE = pulmonary embolism, SGS = simplified Geneva score

**Fig. 4:**
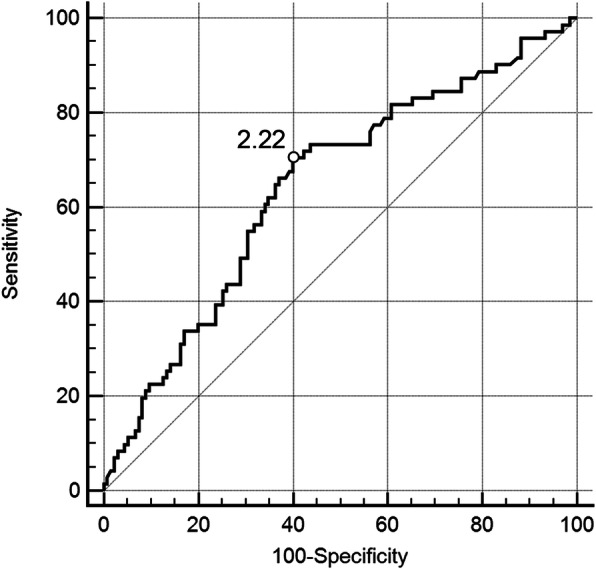
Receiver operating characteristic curve for PE diagnosis by D-dimer evaluation. Patients with SGS ≥ 3SGS ≥ 3 were included (*n* = 206). PE = pulmonary embolism, SGS = simplified Geneva score

To confirm the optimal cutoff point of D-dimer, ROC curve was established. As shown in Fig. 2, patients with D-dimer levels above 1.96 mg/L have a significant risk to suffer from PE (area under curve (AUC), 0.707; 95% CI, 0.678–0.735; *p* < 0.0001). In addition, ROC curves were constructed in patients both with SGS ≤2 and SGS ≥ 3. The results of ROC curve in patients with SGS ≤ 2 were similar to that in all patients, with a D-dimer cutoff point value of 1.96 mg/L (AUC, 0.722; 95% CI, 0.690–0.752; *p <* 0.0001) (Fig. 3). However, in patients with SGS ≥ 3, the D-dimer cutoff point value moved to 2.2 mg/L (AUC, 0.644; 95% CI, 0.574–0.709; *p* = 0.0004) (Fig. 4**)**.

### Sensitivity, specificity, positive predicted value (PPV), and negative predicted value (NPV) of D-dimer cutoff for PE diagnosis

The sensitivity, specificity, positive predicted value (PPV), and negative predicted value (NPV) were calculated for the D-dimer cutoff point value (Table 3). Compared with the rule-out value 0.5 mg/L, the specificity of proposed cutoff point 1.96 mg/L was increased by 0.355 (from 0.328 to 0.683). Meanwhile, the PPV was promoted by 0.105 (from 0.345 to 0.45), while the NPV was only decreased by 0.052 (from 0.884 to 0.832). When combined the SGS assessment and D-dimer detection in PE diagnosis, the D-dimer proposed cutoff point value was changed according to the SGS. In patients with SGS ≥ 3, the D-dimer optimal cutoff point was raised by 0.24 mg/L (from 1.96 mg/L to 2.2 mg/L) compared with that in patients with SGS ≤2.

**Table 3:** Sensitive, specificity, positive predicted value (PPV), and negative predicted value (NPV) of D-dimer for significant PE risk

Group	Proposed D-dimer cutoff point (mg/L)	Sensitivity	Specificity	PPV	NPV
All patients	1.96	0.653	0.683	0.45	0.832
Patients with SGS ≤ 2	1.96	0.628	0.71	0.443	0.838
Patients with SGS ≥ 3	2.22	0.704	0.593	0.476	0.792

*PE* pulmonary embolism, *SGS* simplified Geneva score, *PPV* positive predicted value, *NPV* negative predicted value

## Discussion

It is well investigated that D-dimer test has a good performance in PE ruling-out diagnosis [9, 12, 17]. On the other hand, several studies based on ROC curves have illustrated that high levels of plasma D-dimer (1.96 mg/L in present study) in patients are associated with significant risk for PE incidence [11, 18–20]. However, the best D-dimer cutoff point level for significant PE risk is quite different in these studies. In the first study (PE confirmed patients/study population = 134/370), it was reported that the best D-dimer cutoff level for significant PE risk = should be 2.152 mg/L (AUC, 0.69; 95% CI, 0.64–0.74; *p* < 0.05) [11]. In the second study (PE confirmed patients/study population = 48/544), the optimal D-dimer cutoff point for significant PE risk was determined to be 0.9 mg/L (AUC, 0.76; 95% CI, 0.69–0.82; *p* < 0.001) [18]. In the third study (PE confirmed patients/study population = 40/80), the authors have declared a best D-dimer cutoff point for significant PE risk of 0.83 mg/L (AUC, 0.762; 95% CI, 0.653–0.850; *p <* 0.05) [19]. In the aspect of confirmation of D-dimer cutoff point for significant PE risk, our finding is similar to the first study. Meanwhile, compared with previous studies, our study has the largest investigation population (*n* = 1035) in which the number of PE confirmed patients is 294(28.41%). In the last two studies [18, 19], it is limited in terms of both research population and PE confirmed patients. It was also reported that the age is an important factor while evaluating D-dimer levels for PE diagnosis [21, 22]. But considering that age is one of the SGS items, we did not discuss the age factor independently in our research.

The sensitivity of Geneva score in diagnosis of PE was previously reported as 0.208 in Japanese population [16], which is generally consistence with that in present study (SGS sensitivity, 0.241; data not shown). The specificity of Geneva score for PE diagnosis is relatively high as 0.731 in our study (Data not shown). Combination D-dimer testing and Geneva scores in the diagnosis of PE could improve the diagnostic accuracy and utility of the diagnostic criteria [17, 22]. In our study, the ROC curves were constructed to assess the diagnostic value of D-dimer testing in both PE-likely (SGS ≥ 3) and PE-unlikely (SGS ≤ 2) patients. Our results showed that in patients with SGS ≤ 2, the AUC of the ROC curve raise from 0.328 to 0.722 (comparison with commonly used cutoff point level of 0.5 mg/L). In patients with SGS ≥ 3, the D-dimer optimal cutoff point for significant PE risk moved to the level of 2.2 mg/L, and the PPV increased from 0.345 to 0.476 (comparison with commonly used cutoff point level of 0.5 mg/L).

According to the guidelines for diagnosis and management of acute pulmonary embolism (PE) in 2019, the PPV of D-dimer test is low and D-dimer test is not suggested to applicate in PE confirmation [12]. However, our results revealed that patients with D-dimer levels higher than 1.96 mg/L have a significant risk for PE, in other words, D-dimer may have potential prognostic value in the PE diagnosis. Moreover, lung cancer in patient plays a positive role in PE incidence and poor prognosis [23]. In our study population, there were 68 patients suffered from lung cancer, 28 (41.2%) of which were confirmed with PE. The incidence rate of PE in patients with lung cancer is much higher than 28.4% of the overall study population. Therefore, it is necessary to investigate the prognostic value of D-dimer test for PE diagnosis in patients with lung cancer if large sample size is collected.

In our study, when using the D-dimer commonly used cutoff point (0.5 mg/L) to rule-out PE, 11.6% patients with normal D-dimer levels and confirmed PE would not be excluded by D-dimer testing effectively. Meanwhile, in the patients with PE, 10.9% (32 of 294) had normal levels of D-dimer, which was slightly higher than those reported in most studies, 5.2% (7 of 134) (11), 3.6% (2 of 55) [24], 4% (29 of 725) [25], and 27% (8 of 30) [26]. In present study, we desire to establish optimized test process for PE confirmation through combination SGS pre-test and D-dimer detection in large sample size. CTPA should be our final measure for PE confirmation in patients with high PE risk in our ideal testing process.

There were some limitations in our study. This was a retrospective analysis, and most of the study individuals were hospitalized patient with complex conditions. Plasma D-dimer concentration could be promoted in patients with cancer [27], during pregnancy [28], and in hospitalized patients [29]. Moreover, due to limited sample size of patients with lung cancer, we cannot analyze the D-dimer cut-off point levels for PE diagnosis in this separated population.

## Conclusions

In summary, D-dimer test in combination with SGS pre-test could improve the accuracy of PE diagnosis. Patients with D-dimer levels over 1.96 mg/L (4 times of the normal level) have a significant risk for PE. In addition, in patients with SGS ≥ 3, the D-dimer cutoff point concentration for PE risk moves to the level of 2.2 mg/L. CTPA should be a compulsive measure for PE confirmation in patients with a high level of D-dimer.

## Data Availability

Not applicable.

## Abbreviations

SGS: Simplified Geneva score
PE: Pulmonary embolism;
CTPA: Computed tomographic pulmonary angiogram
ROC: Receiver operating characteristic
YI: Yonden’s index
AUC: Area under curve
ANOVA: Analysis of variance
PPV: Positive predicted value
NPV: Negative predicted value

## References

[1] Goldhaber SZ (2012). Venous thromboembolism: epidemiology and magnitude of the problem. Best Pract Res Clin Haematol.

[2] Raskob GE, Angchaisuksiri P, Blanco AN, Buller H, Gallus A, Hunt BJ, Hylek EM, Kakkar TL, Konstantinides SV, McCumber M, Ozaki Y, Wendelboe A, Weitz JI (2014). ISTH steering Committee for World Thrombosis day. Thrombosis: a major contributor to global disease burden. Semin Thromb Hemost.

[3] Wendelboe AM, Raskob GE (2016). Global burden of thrombosis: epidemiologic aspects. Circ Res.

[4] Keller K, Hobohm L, Ebner M, Kresoja KP, Munzel T, Konstantinides SV, Lankeit M, et al. Eur Heart J. 2019:ehz236. 10.1093/eurheartj/ehz236 31102407

[5] de Miguel-Diez J, Jimenez-Garcia R, Jimenez D, Monreal M, Guijarro R, Otero R, Hernández-Barrera V, Trujillo-Santos J (2014). López de Andrés a, Carrasco-Garrido P. trends in hospital admissions for pulmonary embolism in Spain from 2002 to 2011. Eur Respir J.

[6] Dentali F, Ageno W, Pomero F, Fenoglio L, Squizzato A, Bonzini M (2016). Time trends and case fatality rate of in-hospital treated pulmonary embolism during 11 years of observation in northwestern Italy. Thromb Haemost.

[7] Agarwal S, Clark D, Sud K, Jaber WA, Cho L, Menon V (2015). Gender disparities in outcomes and resource utilization for acute pulmonary embolism hospitalizations in the United States. Am J Cardiol.

[8] Wiener RS, Schwartz LM, Woloshin S (2011). Time trends in pulmonary embolism in the United States: evidence of overdiagnosis. Arch Intern Med.

[9] Harringa JB, Bracken RL, Nagle SK, Schiebler ML, Pulia MS, Svenson JE, Repplinger MD (2017). Negative D-dimer testing excludes pulmonary embolism in non-high risk patients in the emergency department. Emerg Radiol.

[10] Anderson DR, Wells PS (2000). D-dimer for the diagnosis of venous thromboembolism. Curr Opin Hematol.

[11] Sikora-Skrabaka M, Skrabaka D, Ruggeri P, Caramori G, Skoczynski S, Barczyk A (2019). D-dimer value in the diagnosis of pulmonary embolism-may it exclude only?. J Thorac Dis.

[12] Konstantinides SV, Meyer G, Becattini C, Bueno H, Geersing GJ, Harjola VP, Huisman MV, Humbert M, Jennings CS, Jiménez D, Kucher N, Lang IM, Lankeit M, Lorusso R, Mazzolai L, Meneveau N, Ní Áinle F, Prandoni P, Pruszczyk P, Righini M, Torbicki A, Van Belle E, Zamorano JL. ESC Scientific Document Group. 2019 ESC Guidelines for the diagnosis and management of acute pulmonary embolism developed in collaboration with the European Respiratory Society (ERS). Eur Heart J. 2019:ehz405.

[13] Wicki J, Perneger TV, Junod AF, Bounameaux H, Perrier A (2001). Assessing clinical probability of pulmonary embolism in the emergency ward: a simple score. Arch Intern Med.

[14] Klok FA, Mos IC, Nijkeuter M, Righini M, Perrier A, Le Gal G, Huisman MV (2008). Simplification of the revised Geneva score for assessing clinical probability of pulmonary embolism. Arch Intern Med.

[15] Robert-Ebadi H, Mostaguir K, Hovens MM, Kare M, Verschuren F, Girard P, Huisman MV, Moustafa F, Kamphuisen PW, Buller HR, Righini M, Le Gal G (2017). Assessing clinical probability of pulmonary embolism: prospective validation of the simplified Geneva score. J Thromb Haemost.

[16] Ishimaru N, Ohnishi H, Yoshimura S, Kinami S (2018). The sensitivities and prognostic values of the Wells and revised Geneva scores in diagnosis of pulmonary embolism in the Japanese population. Respir Investig.

[17] van Belle A, Buller HR, Huisman MV, Huisman PM, Kaasjager K, Kamphuisen PW, Kramer MH, Kruip MJ, Kwakkel-van Erp JM, Leebeek FW, Nijkeuter M, Prins MH, Sohne M, Tick LW (2006). Christopher Study Investigators. Effectiveness of managing suspected pulmonary embolism using an algorithm combining clinical probability, D-dimer testing, and computed tomography. JAMA.

[18] Shah K, Quaas J, Rolston D, Bansal S, Bania T, Newman D, Wiener D, Lee J (2013). Magnitude of D-dimer matters for diagnosing pulmonary embolus. Am J Emerg Med.

[19] Arnautovic-Torlak V, Pojskic B, Zutic H, Rama A (2014). Values of D-dimer test in the diagnostics of pulmonary embolism. Med Glas (Zenica).

[20] Gao H, Liu H, Li Y (2018). Value of D-dimer levels for the diagnosis of pulmonary embolism: an analysis of 32 cases with computed tomography pulmonary angiography. Exp Ther Med.

[21] Nobes J, Messow CM, Khan M, Hrobar P, Isles C (2017). Age-adjusted D-dimer excludes pulmonary embolism and reduces unnecessary radiation exposure in older adults: retrospective study. Postgrad Med J.

[22] Nagel SN, Steffen IG, Schwartz S, Hamm B, Elgeti T (2019). Age-dependent diagnostic accuracy of clinical scoring systems and D-dimer levels in the diagnosis of pulmonary embolism with computed tomography pulmonary angiography (CTPA). Eur Radiol.

[23] Anagnostopoulos I, Lagou S, Spanorriga MK, Tavernaraki K, Poulakou G, Syrigos KN, Thanos L (2019). Epidemiology and diagnosis of pulmonary embolism in lung cancer patients: is there a role for age adjusted D-dimers cutoff? J Thromb thrombolysis.

[24] Dunn KL, Wolf JP, Dorfman DM, Fitzpatrick P, Baker JL, Goldhaber SZ (2002). Normal D-dimer levels in emergency department patients suspected of acute pulmonary embolism. J Am Coll Cardiol.

[25] Guo Z, Ma Q, Zheng Y, Zhang Y, Ge H (2014). Normal blood D-dimer concentrations: do they exclude pulmonary embolism?. Chin Med J.

[26] Kutinsky I, Blakley S, Roche V (1999). Normal D-dimer levels in patients with pulmonary embolism. Arch Intern Med.

[27] Righini M, Le Gal G, De Lucia S, Roy PM, Meyer G, Aujesky D, Bounameaux H, Perrier A (2006). Clinical usefulness of D-dimer testing in cancer patients with suspected pulmonary embolism. Thromb Haemost.

[28] Chabloz P, Reber G, Boehlen F, Hohlfeld P, de Moerloose P (2001). TAFI antigen and D-dimer levels during normal pregnancy and at delivery. Br J Haematol.

[29] Miron MJ, Perrier A, Bounameaux H, de Moerloose P, Slosman DO, Didier D, Junod A (1999). Contribution of noninvasive evaluation to the diagnosis of pulmonary embolism in hospitalized patients. Eur Respir J.

